# Supplementary material for: Familial t(1;11) translocation is associated with disruption of white matter structural integrity and oligodendrocyte–myelin dysfunction
Source: Mol Psychiatry. 2019 Sep 3;24(11):1641–54. doi: 10.1038/s41380-019-0505-2 (PMC6814440; doi:10.1038/s41380-019-0505-2)
Supplement: Supplementary file 1 — Supplementary Tables and Figure Legends [file 41380_2019_505_MOESM1_ESM.docx]

**SUPPLEMENTARY TABLE AND FIGURES**

**Supplementary Table 1: Clinical characteristics of participants included in this study**

Clinical details of individuals including sex, diagnosis, translocation carrier status, medications at the time of biopsy, age at onset (AAO) and age at sampling (AAS) and reference to the original pedigree previously published in^6,27^.

Extended clinical details:

**Case 1**: Cyclothymic mood changes evident for over 20 years during which time patient experiences low mood, reduced sleep, reduced energy and early morning wakening interspersed with periods of elevated mood and irritability when patient is overactive. These episodes have never warranted admission to psychiatric inpatient care and the episodes of low mood have not persisted for over 2 weeks and the periods of elevated mood have not persisted for more than 4 days.

**Case 2**: First symptoms of major depressive disorder evident at aged 37 years of age. Two episodes of major depression in lifetime, marked by clearly depressed mood, with the duration of the longest episode being 5 months. No manic or psychotic symptoms during either episode.

**Case 3**: Multiple depressive episodes throughout life the first occurring at the age of 39 years. The duration of the longest episode of depression lasted 10 months during which time there was admission to inpatient psychiatric care for 1 month when the patient experiences a severe depressive episode. There have also been frequent cyclothymic episodes with impulsive spending, hyperactivity, reduced sleep for between 1-3 days at any one time. The periods of elevated mood have always been less than 3 days and do not meet the criteria for hypomania.

**Case 4**: First symptoms evident at 13 years of age although formally diagnosed at 20 years of age. Chronic illness course with clear decreasing function. Treatment-resistant schizophrenia. Never free from persecutory delusions and auditory hallucinations.

**Supplementary Table 2: Connectivity remains affected even on excluding Case4**

In order to test for difference in connectivity between Control and t(1;11) carrying affected individuals after exclusion of Case4, statistical analysis was reperformed on remaining individuals.

As shown in the tables, Global Network Degree continues to show a difference (pMCMC=0.0353) while Global Network Strength is not significantly different at a cut-off of p=0.05 (actual pMCMC=0.0684).

**Supplementary Table 3: Comparison of dMRI outputs between affected non-carrier and unaffected control individuals**

Analysis of unaffected controls and the affected non-carrier who carried modifier loci on chr11q2 and chr5q and showed psychiatric symptoms showed no effect on connectivity

**Supplementary Table 4: Comparison of beta-actin (ACTB) mRNA levels across lines using RNA-seq and qPCR**

In order to rule out any effect on ACTB mRNA due to the t(1;11) translocation, ACTB mRNA levels were compared using average FPKM values from RNA-seq and average Ct values from qPCR data generated in this study.

Case line did not show gross changes in either average FPKM or average Ct values when compared with control lines. Average Ct values for GAPDH are also included as a comparison for the variation seen in ACTB.

**Supplementary Figure 1: Characterization of induced pluripotent stem cells from individuals of a large Scottish family carrying a chromosome (1;11) translocation**

(a) Staining of iPS colonies from each of the control and case lines used in this study showed robust expression of NANOG, TRA1-60 and OCT3/4 showing that bona-fide iPS cells were used for deriving oligodendrocytes.

Scale: 50μm

(b) Giemsa banding of a control and a case line clearly show the presence of a (1:11) chromosomal translocation in the cases and a normal karyotype in controls lines used in this study.

**Supplementary Figure 2: Typical X-chromosome inactivation is seen in female lines by H3K27 trimethylation staining**

(a-f) Staining for histone H3 lysine 27 trimethylation shows that female iPSC lines (b,c) show typical X-chromosome inactivation as seen by the presence of nuclear foci (arrowheads) while male lines (a, d-f) show diffuse nuclear staining.

Scale bar (a-f) 10μm

**Supplementary Figure 3: Differential effects of the translocation of proliferation of case-derived OPCs**

(a-c) Breakdown of data from Figure 2d showing quantification of OLIG2+ (a), PDGFRα+ (b) and O4+ (c) in each line. Only Case 3 and Case 4 show statistically significant differences in PDGFRα+ and O4+ percentages (p<0.05 One-way ANOVA with Holm-Sidak’s correction)

(d) Representative images showing fraction of dividing OPCs in each line as visualized by co-staining for PDGFRα+ (green) and the thymidine analog EdU (red). Double-labelled cells are shown by arrows in white.

(e) Quantification of percentage of PDGFRα+ OPCs that co-stain for EdU+ supports previous findings of reduced proliferation in Case3 and Case4 respectively (p<0.05 One-way ANOVA with Holm-Sidak’s correction)

(f) Conversely, Case3 and Case4 lines show premature differentiation that can be seen in cultures as early as 6 days after plate-down (p<0.05 One-way ANOVA with Holm-Sidak’s correction)

Scale bars (d) 50μm

**Supplementary Figure 4: Non-oligodendroglial composition of control and case derived cultures**

1. In addition to oligodendrocytes, cultures at week 3 also contained GFAP positive astrocytes (red) and a small proportion of βIII-tubulin positive (green) neurons.
2. Control lines contained a higher proportion of Ki67+ dividing cells (red) as compared to case derived cells suggesting an increased proportion of glial progenitors in controls even at week3.
3. Quantification of week 3 cultures showed equivalent proportion of GFAP+ astrocytes and TUJ1+ neurons between control and case lines but an increased proportion of Ki67+ progenitors in controls (Controls: 12.30% ±2.8, Cases: 5.54% ±1.2, p<0.05 unpaired t-test).
4. A decrease in full-length DISC1 expression could be seen in case iPSCs as well as case-derived OPCs (Controls: 0.72±0.10 vs Cases: 0.22±0.05, p<0.05 One-way ANOVA with Holm-Sidak’s correction)

Scale bars (a,b) 50μm

**Supplementary Figure 5: Distribution of in-vitro morphology and in-vivo myelin segment changes in case-derived oligodendrocytes**

1. Representative images of O4 staining of individual lines
2. Quantification of cell area shows a significant decrease in cell area as quantified from O4 staining for Case2, Case3 and Case4 but not Case1 (p<0.05 One-way ANOVA with Holm-Sidak’s correction)

(c,d) Internodal segment lengths are decreased in both Case2 and Case4-derived OPCs transplanted into *MBP^shi/shi^; Rag2^-/-^* neonatal cortices at 8 and 13-weeks post-transplant (8 weeks: 202(3), 79(3), 121(3) and 199(3) segments measured respectively; 13 weeks: 183(1), 86(2), 113(3) and 32(3) segments measured respectively, brackets indicate animals analysed, p<0.05 One-way ANOVA with Holm-Sidak’s correction)

(e) Frequency histogram showing that myelin sheaths formed by Case-derived oligodendrocytes are predominantly shorter than control derived cells.

Scale bars: (a) 10μm

**Supplementary Figure 6: Expression of DISC1CP1 affects expression of full-length DISC1 suggesting a dominant-negative mechanism**

Variable amounts of FLAG tagged full-length DISC1 expressing construct was co-expressed with a construct expressing the chimeric protein DISC1CP1 in human MO3.13 cell line.

Expression of DISC1CP1 decreased expression of full-length DISC1 as visualized using an antibody against the C-terminal of DISC1 (upper panel) as well as antibody against the FLAG tag (middle panel).

Beta-actin (lower panel) was used as a loading control.
