# Supplementary material for: Familial t(1;11) translocation is associated with disruption of white matter structural integrity and oligodendrocyte–myelin dysfunction
Source: Mol Psychiatry. 2019 Sep 3;24(11):1641–54. doi: 10.1038/s41380-019-0505-2 (PMC6814440; doi:10.1038/s41380-019-0505-2)
Supplement: Supplementary file 2 — Supplementary Methods [file 41380_2019_505_MOESM2_ESM.docx]

**DYSFUNCTIONAL MYELIN DEVELOPMENT IN MAJOR MENTAL ILLNESS**

**Vasistha et al.,**

**Supplementary Methods**

**Connectome Analysis- dMRI acquisition**

All imaging data were collected on a Siemens Magnetom Verio 3T MRI scanner running Syngo MR B17 software (Siemens Healthcare, Erlangen, Germany). For each subject, whole brain diffusion MRI (dMRI) data were acquired using a single- shot spin-echo echo-planar (EP) imaging sequence with diffusion-encoding gradients applied in 56 directions (b=1000 s/mm2) and six T2-weighted (b=0 s/mm2) baseline scans. Fifty-five 2.5 mm thick axial slices were acquired with a field-of-view of 240 mm and matrix 96 × 96 giving 2.5 mm isotropic voxels. In the same session, a 3D T1- weighted inversion recovery-prepared fast spoiled gradient-echo (FSPGR) volume was acquired in the coronal plane with 160 contiguous slices and 1 mm isotropic voxel resolution.

Image processing

Each 3D T1-weighted FSPGR volume was parcellated into 85 cortical (34 per hemisphere) and sub-cortical (eight per hemisphere) regions-of-interest (ROI), plus the brain stem, using the Desikan-Killiany atlas in FreeSurfer (http://surfer.nmr.mgh.harvard.edu) (1). The results of the segmentation procedure were then used to construct grey and white matter masks for use in network construction and to constrain the tractography output as described below. Using tools provided by the FDT package in FSL (http://fsl.fmrib.ox.ac.uk/fsl), the dMRI data were pre-processed to reduce systematic imaging distortions and bulk subject motion artifacts by affine registration of all subsequent EP volumes to the first T2- weighted EP volume (2). Skull stripping and brain extraction were performed on the registered T2-weighted EP volumes and applied to the fractional anisotropy (FA) volume calculated by DTIFIT in each subject (3,4). The neuroanatomical ROIs determined by Freesurfer were then aligned from 3D T1-weighted volume to diffusion space using a cross-modal nonlinear registration method. As a first step, linear registration was used to initialize the alignment of each brain-extracted FA volume to the corresponding FreeSurfer extracted 3D T1-weighted brain volume using a mutual information cost function and an affine transform with 12 degrees of freedom (5). Following this initialization, a nonlinear deformation field based method (FNIRT) was used to refine local alignment (6). FreeSurfer segmentations and anatomical labels were then aligned to diffusion space using nearest neighbour interpolation.

**Tractography**

Whole-brain probabilistic tractography was performed using FSL’s BedpostX/ProbTrackX algorithm (7,8). Probability density functions, which describe the uncertainty in the principal directions of diffusion, were computed with a two-fibre model per voxel (8). Streamlines were then constructed by sampling from these distributions during tracking using 100 Markov Chain Monte Carlo iterations with a fixed step size of 0.5 mm between successive points. Tracking was initiated from all white matter voxels and streamlines were constructed in two collinear directions until terminated by the following stopping criteria designed to minimize the amount of anatomically implausible streamlines: (i) exceeding a curvature threshold of 70 degrees; (ii) entering a voxel with FA below 0.1 (9); (iii) entering an extra-cerebral voxel; (iv) exceeding 200 mm in length; and (v) exceeding a distance ratio metric of 10. The distance ratio metric (10), excludes implausibly tortuous streamlines. For instance, a streamline with a total path length 10 times longer than the distance between end points was considered to be invalid. The values of the curvature, anisotropy and distance ratio metric constraints were set empirically and informed by visual assessment of the resulting streamlines.

**Network construction**

FA-weighted networks were constructed by recording the mean FA value along streamlines connecting all ROI (network node) pairs. The endpoint of a streamline was considered to be the first grey matter ROI encountered when tracking from the seed location. As tractography is prone to producing false connections (11), prior knowledge of white matter anatomy was used to discard a proportion of spurious connections. Specifically, any implausible streamlines traversing from one cortical hemisphere to any contralateral subcortical node were discarded (12). Self- connections were removed, and if no streamlines were found between a pair of nodes, the corresponding matrix entry was set to zero. To reduce spurious connections further, a two-step threshold on the network FA weights was then applied: (i) for each subject, discard the weakest 25% of weights in the matrix by connection probability; (ii) across the cohort, only retain connections which occur in at least 50% of subjects. The second step is required to discard connections which have been removed for some subjects, but not others, by the first step. Finally, for each FA-weighted connectivity matrix, four global network measures were then

3 computed using the brain connectivity toolbox (13; https://sites.google.com/site/bctnet), namely, network degree (number of links per node), strength (the average sum of weights per node), clustering coefficient (fraction of triangles around a node) and efficiency (the average of the inverse shortest path length).

**References**

1. Desikan RS, Ségonne F, Fischl B, Quinn BT, Dickerson BC, Blacker D, Buckner RL, Dale AM, Maguire RP, Hyman BT, Albert MS, Killiany RJ. An automated labeling system for subdividing the human cerebral cortex on MRI scans into gyral based regions of interest. NeuroImage 2006;31:968–980.

2. Jenkinson M, Smith S. A global optimisation method for robust affine registration of brain images. Med. Image Anal. 2001;5:143–156.

3. Smith SM. Fast robust automated brain extraction. Hum. Brain Mapp. 2002;17:143–155.
4. Basser PJ, Pierpaoli C. Microstructural and physiological features of tissues elucidated by quantitative-diffusion-tensor MRI. J. Magn. Reson. B 1996;111:209–219.

5. Jenkinson M, Smith S. A global optimisation method for robust affine registration of brain images. Med. Image Anal. 2001;5:143–156.

6. Andersson JLR, Jenkinson M, Smith S. Non-linear registration, aka Spatial normalisation. 2007. Technical Report TR07JA2, Oxford Centre for Functional Magnetic Resonance Imaging of the Brain, University of Oxford.

7. Behrens TE, Woolrich MW, Jenkinson M, Johansen-Berg H, Nunes RG, Clare S, Matthews PM, Brady JM, Smith SM. Characterization and propagation of uncertainty in diffusion-weighted MR imaging. Magn. Reson. Med. 2003;50:1077–1088.
8. Behrens TE, Johansen-Berg H, Jbabdi S, Rushworth MFS, Woolrich MW. Probabilistic diffusion tractography with multiple fibre orientations: What can we gain? NeuroImage 2007;34:144–155.

9. Verstraete E, Veldink JH, Mandl RCW, Van Den Berg LH, Van Den Heuvel MP. Impaired structural motor connectome in amyotrophic lateral sclerosis. PLoS One 2011;6:e24239.
10. Bullitt E, Gerig G, Pizer SM, Lin W, Aylward SR. Measuring tortuosity of the intracerebral vasculature from MRA images. IEEE Trans. Med. Imaging 2003;22:1163–1171.

11. Van Essen DC, Ugurbil K. The future of the human connectome. NeuroImage 2012;62:1–12.

12. Funnell MG, Corballis PM, Gazzaniga MS. Cortical and subcortical interhemispheric interactions following partial and complete callosotomy. Arch. Neurol. 2000;57:185–189.
13. Rubinov M, Sporns O. Complex network measures of brain connectivity: uses and interpretations. NeuroImage 2010;52:1059–1069.

**iPSC conversion to OPCs**

hiPSCs were cultured in Matrigel coated plates in E8 medium (Life Technologies). For neuralization iPSCs were lifted using a mix of dispase and collagenase (Life Technologies) and cultured for 7 days, with dual-SMAD inhibition (10µM SB431542 and 10nM LDN193189) in chemically defined media (CDM) that contained 50% Iscove’s modified Dulbecco’s medium (Invitrogen, 50% F12, BSA (5 mg/ml, Europa), 1% chemically defined Lipid 100 (Invitrogen), monothioglycerol (Sigma- Aldrich), insulin (7mg/ml, Roche, Basel), transferrin (15 mg/ml, Roche), 1% penicillin/streptomycin), supplemented with N-acetyl cysteine (1 mM, Sigma), activin Inhibitor (10 µM, R&D Systems), and Dorsomorphin (2 µM, Merck Millipore). The resulting neurospheres were cultured in suspension with media changes every 2–3 days. Subsequently, neurospheres﻿ were plated on laminin (Sigma Aldrich) coated 6-well plates for 2 days to induce neural rosette formation. Rosettes were picked and expanded before being ventralized by adding sonic hedgehog agonist purmorphamine (1 µM, Calbiochem) for 7 days in Advanced DMEM/F12 (Invitrogen) containing: 1% N-2 supplement (Invitrogen), 1% B27 supplement (Invitrogen), 1% penicillin/streptomycin (Invitrogen), 0.5% GlutaMAX (Invitrogen), and 5 µg/ml heparin (Sigma). Ventral patterned progenitor cells were then further expanded in the presence of basic fibroblast growth factor (FGF-2) (10 ng/ml, PeproTech) for 7 days leading to the formation of spheres that were cultured as a suspension. Differentiation of these progenitor cells was induced by culturing in the same medium but without FGF2 for a further 2 weeks. Spheres at this stage were enriched for OLIG2+ cells and called as Oligospheres. Oligospheres were further expanded for 2 weeks in media containing FGF2 (10 ng/ml), PDGFa (Platelet-derived growth factor alpha, 20 ng/ml, PeproTech), purmorphamine (1 µM), and SAG (1 µM, Calbiochem), IGF-1 (10 ng/ml, PeproTech), T3 (60 ng/ml, Sigma), and Insulin-Transferring-Sodium Selenite ITS (Gibco) before dissociation to obtain OPCs.

**RNA sequencing analysis**

**Library Preparation**

Libraries were prepared from each total-RNA sample using the TruSeq Stranded Total RNA with Ribo-Zero kit (#RS-122-2201) according to the provided protocol.

100ng of total-RNA was processed to deplete rRNA before being purified, fragmented and primed with random hexamers. Primed RNA fragments were reverse transcribed into first strand cDNA using reverse transcriptase and random primers. RNA templates were removed and a replacement strand synthesised incorporating dUTP in place of dTTP to generate ds cDNA. AMPure XP beads (Beckman Coulter, #A63881) were then used to separate the ds cDNA from the second strand reaction mix, providing blunt-ended cDNA. A single 'A' nucleotide was added to the 3' ends of the blunt fragments to prevent them from ligating to another during the subsequent adapter ligation reaction, and a corresponding single 'T' nucleotide on the 3' end of the adapter provided a complementary overhang for ligating the adapter to the fragment. Multiple indexing adapters were then ligated to the ends of the ds cDNA to prepare them for hybridisation onto a flow cell, before 15 cycles of PCR were used to selectively enrich those DNA fragments that had adapter molecules on both ends and amplify the amount of DNA in the library suitable for sequencing. After amplification libraries were purified using AMPure XP beads.

**Library QC**

Libraries were quantified by fluorometry using the Qubit dsDNA HS assay and assessed for quality and fragment size using the Agilent Bioanalyser with the DNA HS Kit (#5067-4626). Due to the presence of leftover adapters an extra round of purification with AMPure XP beads was undertaken and libraries were again quantified with the Qubit dsDNA HS assay and assessed using the Agilent Bioanalyser.

**Sequencing**

Sequencing was performed using the NextSeq 500/550 High-Output v2 (150 cycle) Kit (# FC-404-2002) on the NextSeq 550 platform (Illumina Inc, #SY-415-1002). 2

Libraries were combined in equimolar pools as requested by the investigator (based on the qPCR quantification results) and run across 3 High-Output Flow Cells.

**Analysis**

Three biological replicates were sequenced for each of 3 case and 2 control lines. However, one control replicate was discarded due to low quality sequencing data, leaving 9 case and 5 control samples. Raw reads in FASTQ format were mapped to the human genome using version 2.4.0i of the STAR RNA-seq aligner [1] and per-gene read counts summarised using featureCounts version 1.4.6-p2 [2] (genome sequences and annotations were downloaded from Ensembl [3], version 86). Differential expression was performed for all case versus control samples using DESeq2 version 1.16.1 [4]. Enrichment analyses were performed with topGO version 2.28.0 [5] and the camera method [6] from limma version 3.32.10 [7]. A multiple testing correction was used based on false discovery rate (FDR) of 0.05.

**References**

[1] Dobin, A. et al., STAR: ultrafast universal RNA-seq aligner. Bioinformatics 29 (1), 15–21 (2013).

[2] Liao, Y., Smyth, G. K. & Shi, W., featureCounts: an efficient general purpose program for assigning sequence reads to genomic features. Bioinformatics 30 (7), 923–930 (2014).

[3] Zerbino, D. R. et al., Ensembl 2018. Nucleic Acids Reseach 46 (D1), D754–D761 (2018)

[4] Love, M. I., Huber, W. & Anders, S. Moderated estimation of fold change and dispersion for RNA-seq data with DESeq2. Genome Biol. 15, 550 (2014).

[5] Alexa, A., Rahnenführer J. & Lengauer T., Improved scoring of functional groups from gene expression data by decorrelating GO graph structure. Bioinformatics 22 (13), 1600-7 (2006).

[6] Wu D. and Smyth G. K., Camera: a competitive gene set test accounting for inter-gene correlation. Nucleic Acids Research, 40 (17), e133 (2012).

[7] Ritchie M. E. et al., limma powers differential expression analyses for RNA-sequencing and microarray studies. Nucleic Acids Research, 43 (7), pp. e47 (2015).

**MO3.13 cell line**

The MO3.13 cell line was a generous gift from Prof. Neil Cashman, University of British Columbia^1^. The cell line was maintained in Advanced DMEM/F12 (Life Technologies) supplemented with 10% foetal calf serum and 2mM L-glutamine (Life Technologies).

**Plasmids**

FLAG tagged full-length DISC1 (FLAG-DISC1) and DISC1 chimeric protein (DISC1CP1) expressing plasmids have been described previously^2^**.** For EGFP expression, pEGFP-C1 plasmid was obtained from Clontech (Clontech Inc.). Plasmids were purified from bacterial cells grown overnight in LB media with appropriate antibiotics (QIAGEN Maxiprep kit). Cells were transfected using variable amounts of the plasmids with Lipofectamine (ThermoFisher).

**Western blot analysis**

**﻿**Transfected MO3.13 cells were lysed in protease inhibitor (Roche) containing RIPA Buffer and protein concentration quantified using a bicinchoninic acid assay kit.

Equal amounts of protein were loaded on pre-cast 4-16% Tris-Glycine polyacrylamide gels (ThermoFisher) beside PageRuler (ThermoFisher) pre-stained protein ladder and run at room temperature for 40 minutes at 200V. The gel was then blotted onto a PVDF membrane pre-activated in methanol at room temperature for 1 hour at 20V.

In order to detect proteins of interest, the membrane was removed and blocked in 5% milk protein solution in 0.1% Tween-20 containing phosphate buffered-saline. Primary antibodies were added overnight at 4°C. After washes the next day, the membrane was incubated with HRP conjugated secondary antibodies (1:5000, Jackson Immunoresearch) for 2 hours at room temperature and developed using chemiluminescence based detection kits (Amersham)

The following antibodies were used: rabbit anti-DISC1 (Proteintech 15500-1-AP 1/1000), mouse anti-FLAG (Sigma F3165 1/10,000) and mouse anti- ßactin (1/5000).

1 McLaurin J, Trudel GC, Shaw IT, Antel JP, Cashman NR. A human glial hybrid cell line differentially expressing genes subserving oligodendrocyte and astrocyte phenotype. *J Neurobiol* 1995; **26**: 283–293.

2 Eykelenboom JE, Briggs GJ, Bradshaw NJ, Soares DC, Ogawa F, Christie S *et al.* A t(1;11) translocation linked to schizophrenia and affective disorders gives rise to aberrant chimeric DISC1 transcripts that encode structurally altered, deleterious mitochondrial proteins. *Hum Mol Genet* 2012; **21**: 3374–3386.
