# Supplementary material for: Familial t(1;11) translocation is associated with disruption of white matter structural integrity and oligodendrocyte–myelin dysfunction
Source: Mol Psychiatry. 2019 Sep 3;24(11):1641–54. doi: 10.1038/s41380-019-0505-2 (PMC6814440; doi:10.1038/s41380-019-0505-2)
Supplement: Supplementary file 3 — Supplementary Figure-Pedigree [file 41380_2019_505_MOESM3_ESM.pdf]

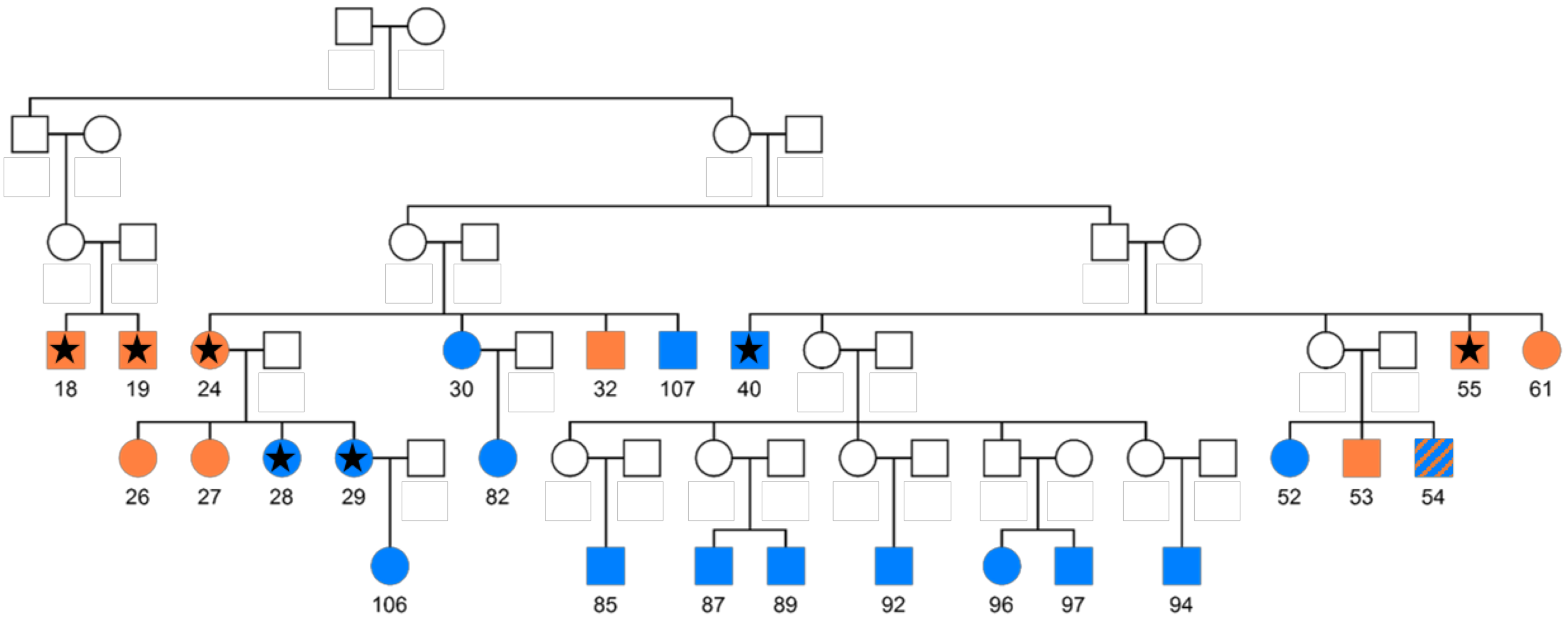

Key:
Orange= translocation carrier
Blue= translocation non-carrier
Cross-hatched blue-orange= affected non-carrier
Star= individuals from whom iPSC were derived for study
