# Supplementary material for: Familial t(1;11) translocation is associated with disruption of white matter structural integrity and oligodendrocyte–myelin dysfunction
Source: Mol Psychiatry. 2019 Sep 3;24(11):1641–54. doi: 10.1038/s41380-019-0505-2 (PMC6814440; doi:10.1038/s41380-019-0505-2)
Supplement: Supplementary file 4 — Supplementary Table 1 [file 41380_2019_505_MOESM4_ESM.pdf]

GLOBAL NETWORK DEGREE (WITHOUT Case4)

	POST. MEAN	LOWER 95% CI	UPPER 95% CI	EFF.SAMP	pMCMC
(INTERCEPT)	1.02438	0.36225	2.25510	3911	0.1216
DIAGNOSIS	-1.10485	2.16010	-0.11928	8673	**0.0353***
AGE	0.01203	-0.01651	0.04085	3394	0.3880

GLOBAL NETWORK STRENGTH (WITHOUT Case4)

	POST. MEAN	LOWER 95% CI	UPPER 95% CI	EFF.SAMP	pMCMC
(INTERCEPT)	1.630103	0.361645	2.942390	8054	0.0151
DIAGNOSIS	-0.925860	-1.880168	0.124224	9000	**0.0684**
AGE	-0.009228	-0.034927	0.016131	9338	0.4664
