# Supplementary material for: Familial t(1;11) translocation is associated with disruption of white matter structural integrity and oligodendrocyte–myelin dysfunction
Source: Mol Psychiatry. 2019 Sep 3;24(11):1641–54. doi: 10.1038/s41380-019-0505-2 (PMC6814440; doi:10.1038/s41380-019-0505-2)
Supplement: Supplementary file 5 — Supplementary Table 2 [file 41380_2019_505_MOESM5_ESM.pdf]

COMPARISON OF dMRI OUTPUTS BETWEEN AFFECTED NON-CARRIER AND UNAFFECTED CONTROLS

GLOBAL STRENGTH

	POST.MEAN	L-95% CI	U-95% CI	EFF.SAMP	pMCMC
(INTERCEPT)	1.332515	4.057603	1.305672	6216	0.300
DIAGNOSIS	1.466006	0.943506	3.895556	4287	0.206
AGE	0.007346	0.043385	0.026525	9000	0.635

GLOBAL DEGREE

	POST.MEAN	L-95% CI	U-95% CI	EFF.SAMP	pMCMC
(INTERCEPT)	-1.29842	-3.82651	1.15863	5706	0.275
DIAGNOSIS	0.48255	-1.61269	2.71490	5002	0.623
AGE	0.02338	-0.00873	0.05693	9000	0.148

CLUSTERING COEFFICIENT

	POST.MEAN	L-95% CI	U-95% CI	EFF.SAMP	pMCMC
(INTERCEPT)	-1.240372	-3.420873	1.085381	7379	0.2596
DIAGNOSIS	1.892257	-0.036190	3.867386	6388	0.0613
AGE	-0.023539	-0.052909	0.007493	9000	0.1147

GLOBAL EFFICIENCY

	POST.MEAN	L-95% CI	U-95% CI	EFF.SAMP	pMCMC
(INTERCEPT)	-0.569204	-2.968351	1.939521	5980	0.609
DIAGNOSIS	1.256684	-0.791421	3.576169	4008	0.233
AGE	-0.023495	-0.055236	0.008389	9000	0.142
