# Supplementary material for: Familial t(1;11) translocation is associated with disruption of white matter structural integrity and oligodendrocyte–myelin dysfunction
Source: Mol Psychiatry. 2019 Sep 3;24(11):1641–54. doi: 10.1038/s41380-019-0505-2 (PMC6814440; doi:10.1038/s41380-019-0505-2)
Supplement: Supplementary file 6 — Supplementary Table 3 [file 41380_2019_505_MOESM6_ESM.pdf]

SUPPLEMENTARY TABLE 3: CASE DESCRIPTIONS AND CELL LINE IDs USED IN THIS STUDY

**Subject**	**Sex**	**Diagnosis**	**t(1:11) Translocation status**	**Psychotropic Medications (At time of biopsy)**	**AAO/AAS**	**Pedigree Reference (Ryan et al., 2018)**	**Pedigree Reference (Blackwood et al., 2001)**
Control 1	Male	Unaffected	Non-Carrier	None	NA/73	40	IV 15
Control 2	Female	Unaffected	Non-Carrier	None	NA/52	28	not included*
Control 3	Female	Unaffected	Non-Carrier	None	NA/46	29	not included*
Case 1	Male	Cyclothymia	Carrier	None	45/66	55	not included*
Case 2	Male	Major Depressive Disorder	Carrier	Fluoxetine	37/75	24	IV 11
Case 3	Male	Major Depressive Disorder	Carrier	Citalopram	39/58	19	IV 9
Case 4	Male	Schizophrenia	Carrier	Sodium valproate, Clozapine, Lithium Carbonate	20/60	18	IV 8

* Not included in the pedigree in Blackwood et al., 2001
