# Supplementary figures and images for: Familial t(1;11) translocation is associated with disruption of white matter structural integrity and oligodendrocyte–myelin dysfunction
Source: Mol Psychiatry. 2019 Sep 3;24(11):1641–54. doi: 10.1038/s41380-019-0505-2 (PMC6814440; doi:10.1038/s41380-019-0505-2)

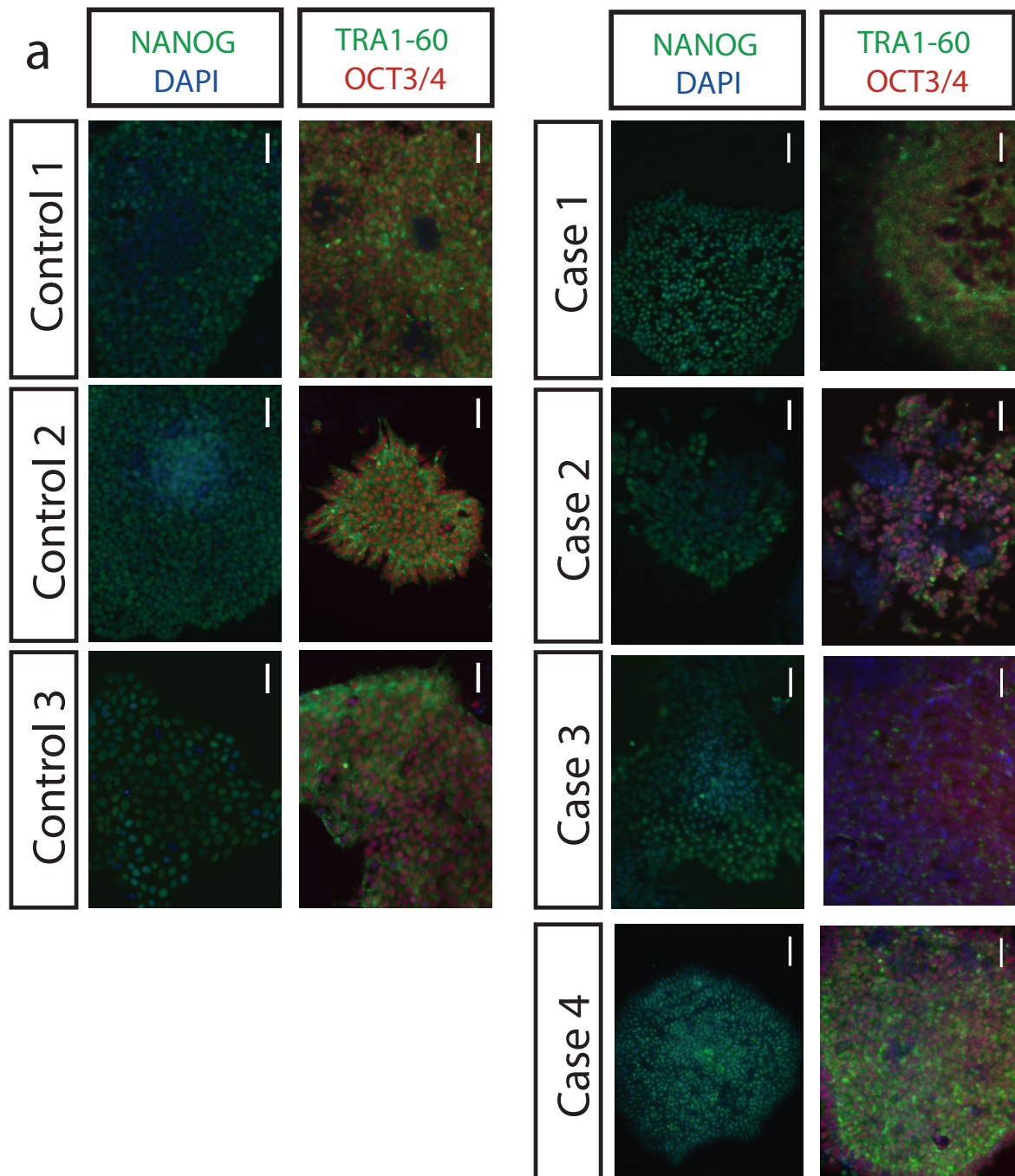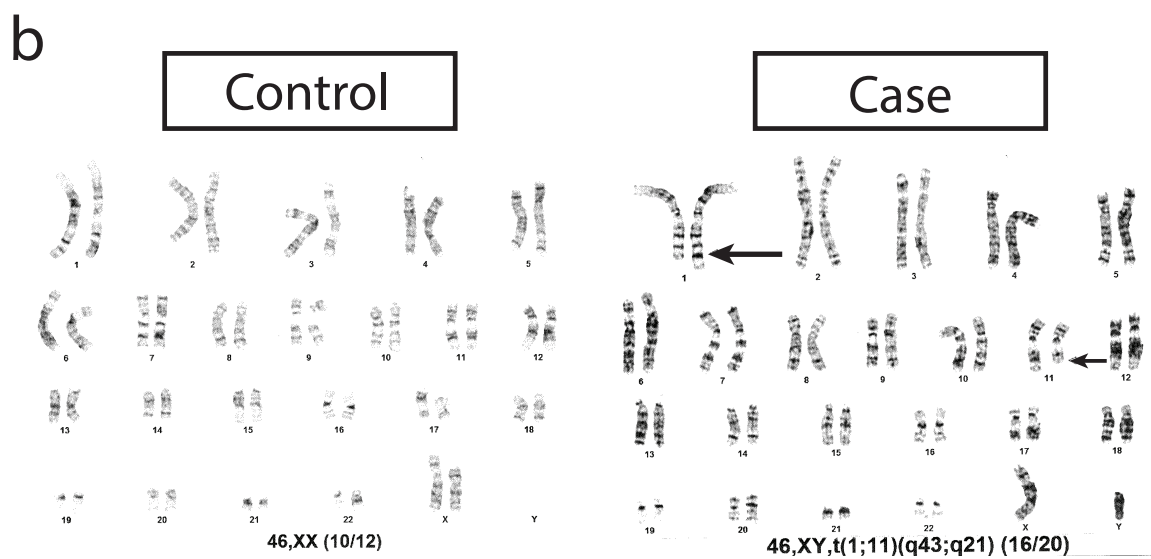

Supplement: Supplementary file 8 — Supplementary Fig. 1 [file 41380_2019_505_MOESM8_ESM.pdf]

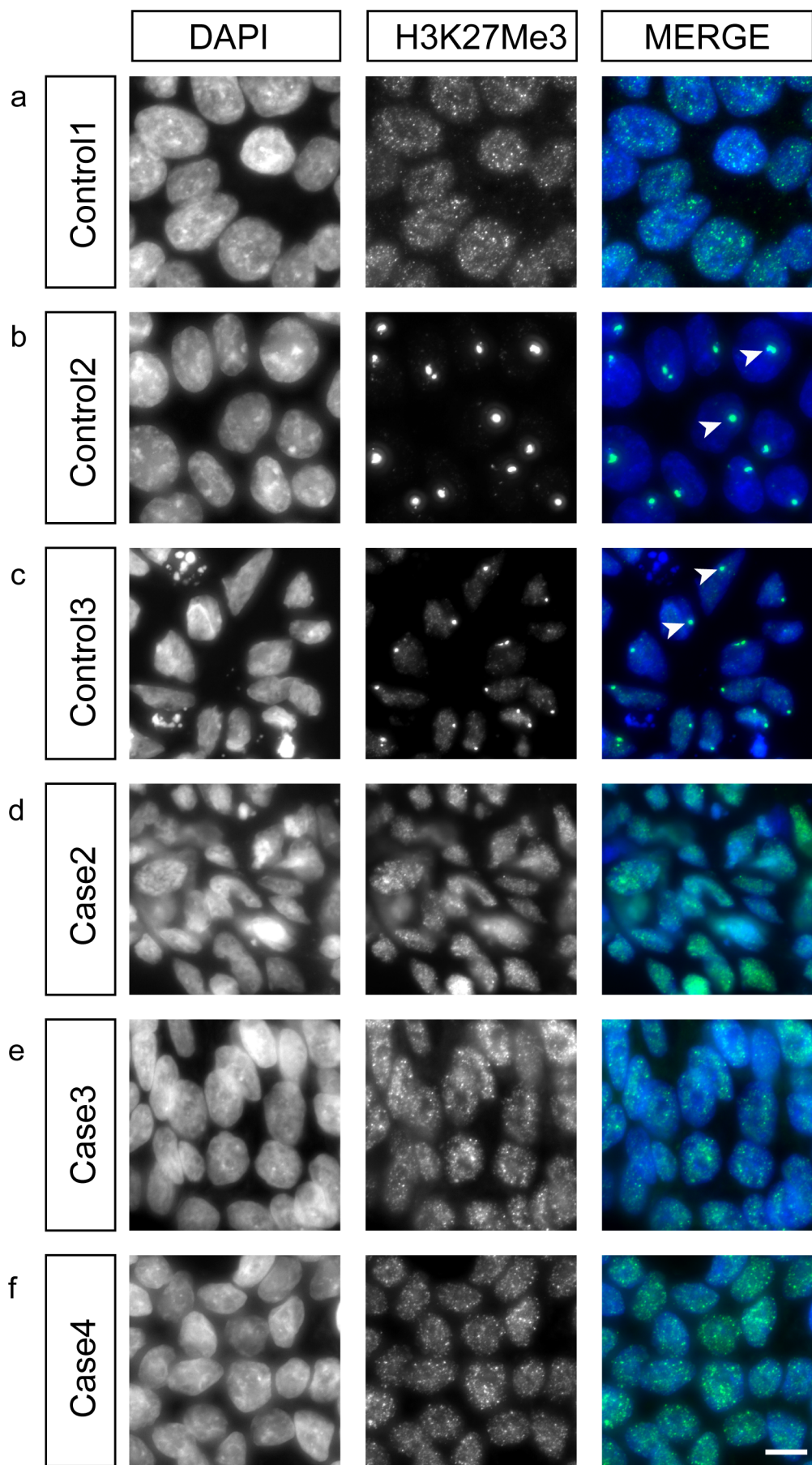

Supplement: Supplementary file 9 — Supplementary Fig. 2 [file 41380_2019_505_MOESM9_ESM.pdf]

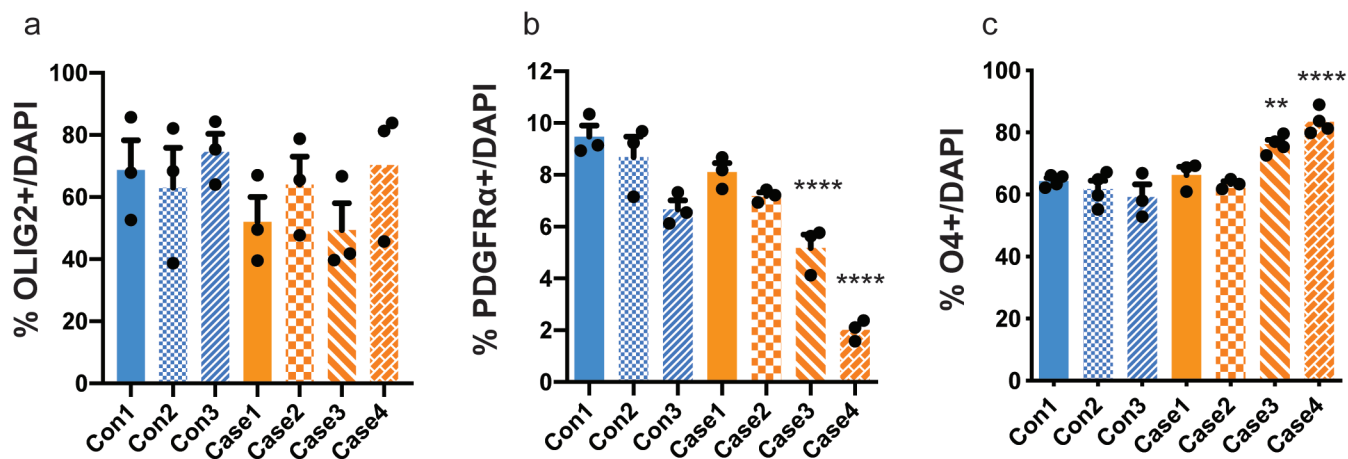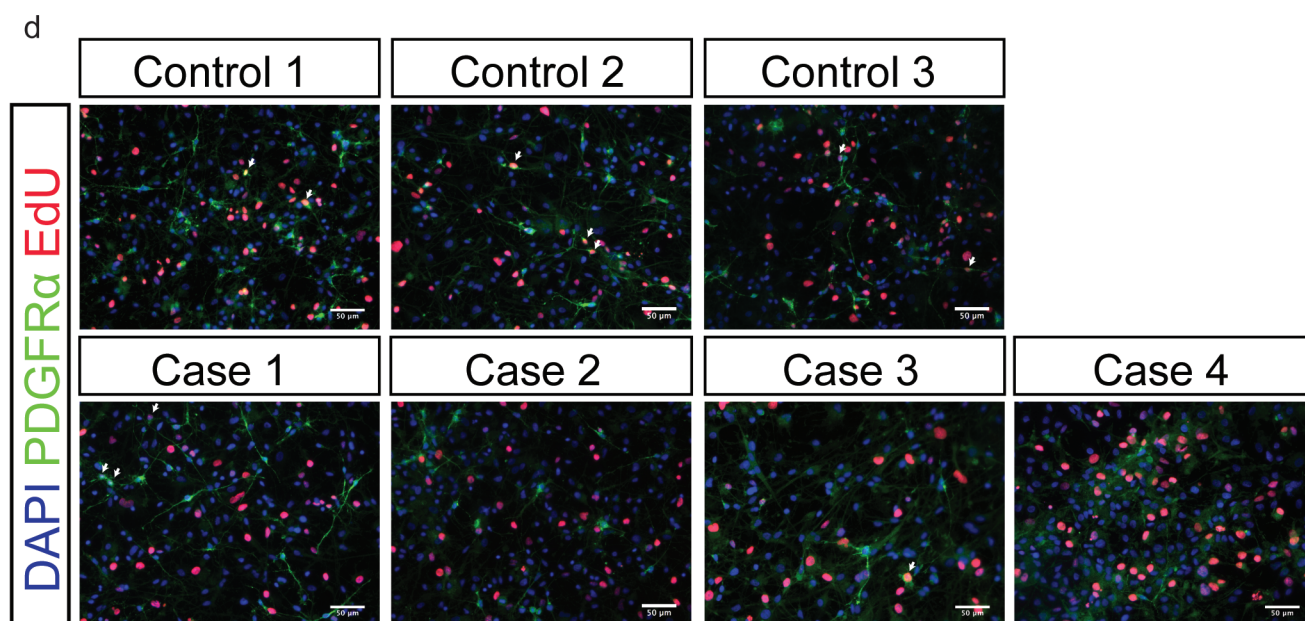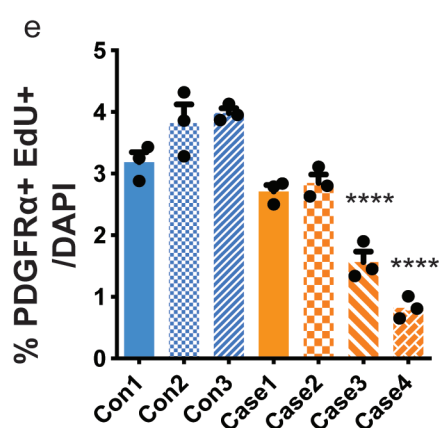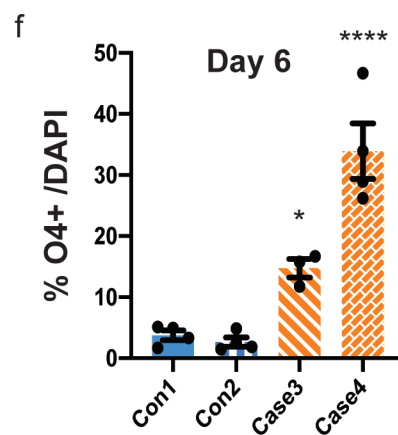

Supplement: Supplementary file 10 — Supplementary Fig. 3 [file 41380_2019_505_MOESM10_ESM.pdf]

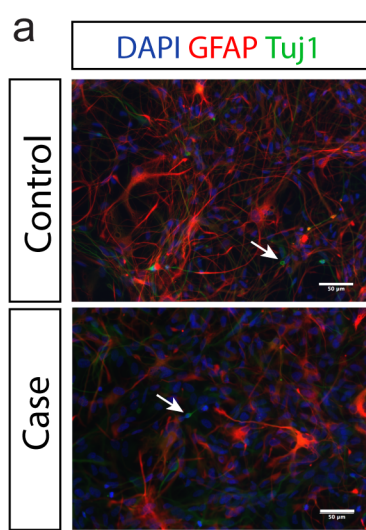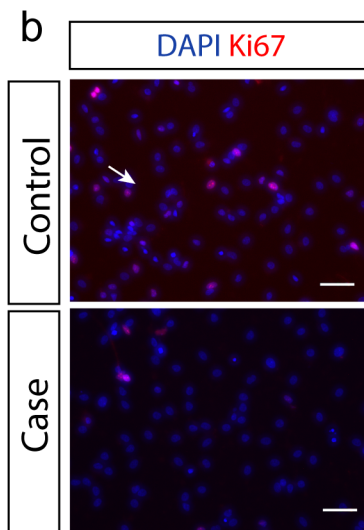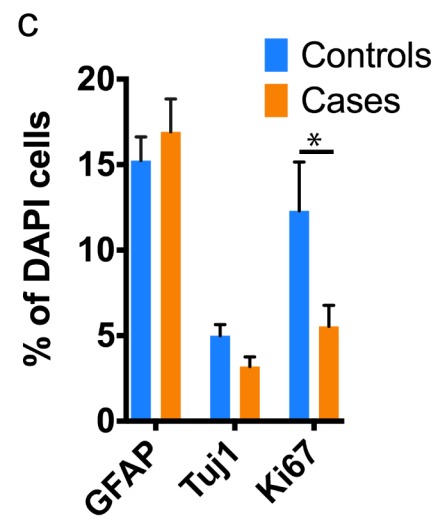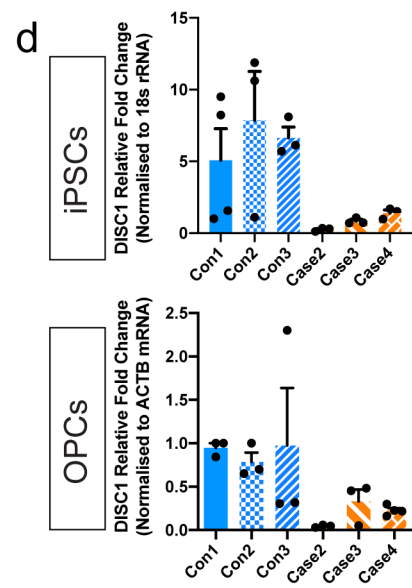

Supplement: Supplementary file 11 — Supplementary Fig. 4 [file 41380_2019_505_MOESM11_ESM.pdf]

a

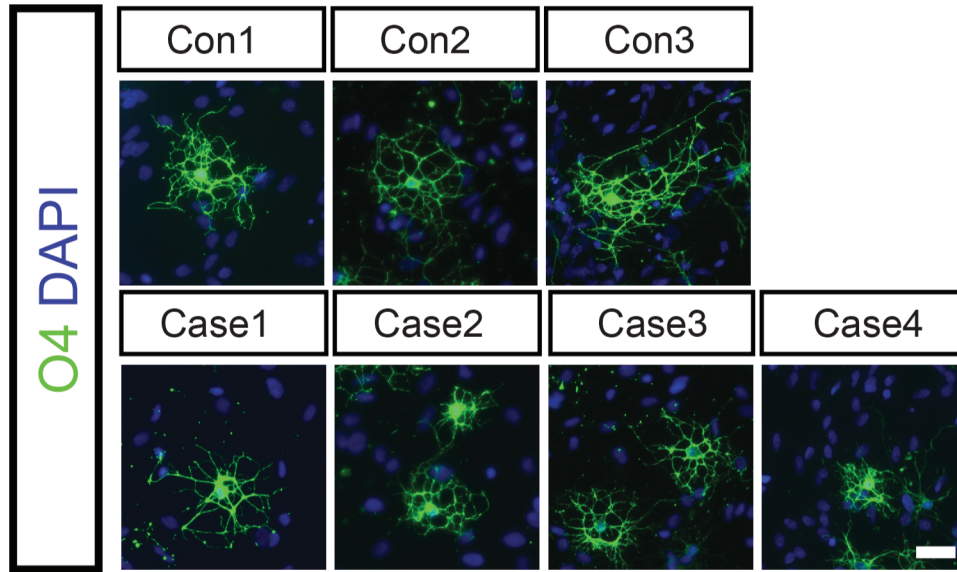

b

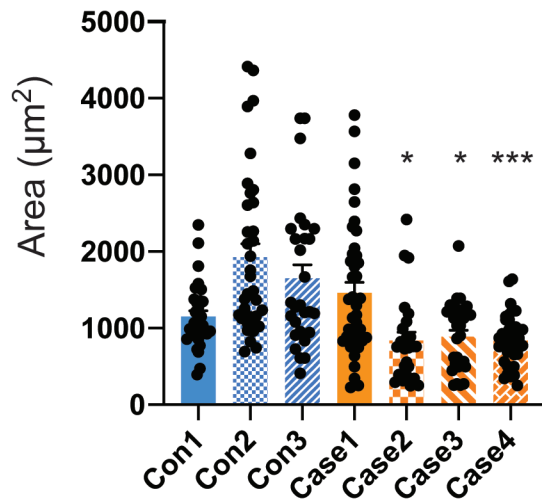

c

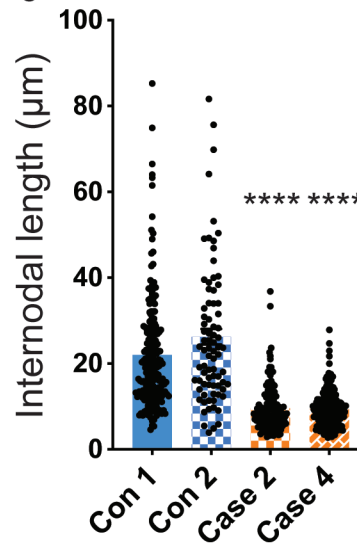

d

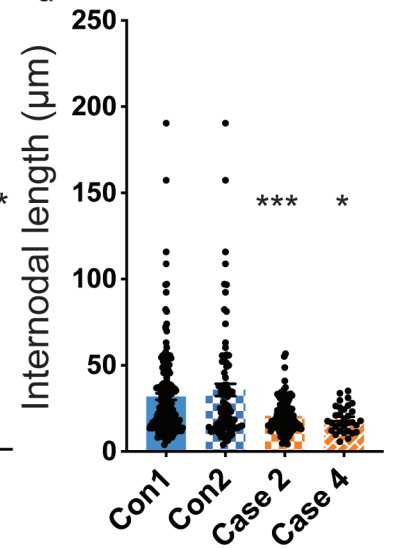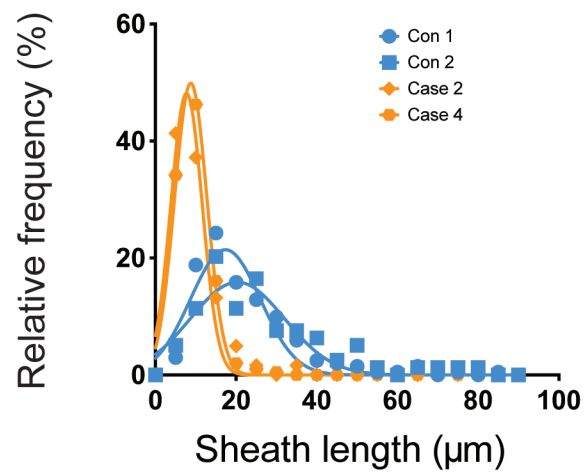

Supplement: Supplementary file 12 — Supplementary Fig. 5 [file 41380_2019_505_MOESM12_ESM.pdf]
